# Comparison of Mitochondrial Respiration in M. triceps brachii and M. vastus lateralis Between Elite Cross-Country Skiers and Physically Active Controls

**DOI:** 10.3389/fphys.2019.00365

**Published:** 2019-04-05

**Authors:** Jonathan Berg, Vidar Undebakke, Øystein Rasch-Halvorsen, Lars Aakerøy, Øyvind Sandbakk, Arnt Erik Tjønna

**Affiliations:** ^1^Department of Circulation and Medical Imaging, Norwegian University of Science and Technology, Trondheim, Norway; ^2^Clinic of Thoracic and Occupational Medicine, St. Olav’s University Hospital, Trondheim, Norway; ^3^Centre for Elite Sports Research, Department of Neuromedicine and Movement Science, Norwegian University of Science and Technology, Trondheim, Norway

**Keywords:** cross-country skiing, endurance athletes, high-resolution respirometry, mitochondria, oxidative phosphorylation, peak oxygen uptake, upper-body

## Abstract

**Rationale:**

The main purposes of this study were to compare mitochondrial respiration in M. triceps brachii and M. vastus lateralis between elite cross-country (XC) skiers and physically active controls (CON), and to explore the associations between mitochondrial respiration in these muscles and peak oxygen uptake ($\dot{V}$O_2peak_) in arm- and leg-dominant exercise modes.

**Methods:**

Thirteen male elite XC skiers (age: 25 ± 4; peak oxygen uptake ($\dot{V}$O_2peak_): 75.5 ± 4.2 mL⋅kg^-1^⋅min^-1^) and twelve CON (age: 26 ± 3; $\dot{V}$O_2peak_: 57.2 ± 6.4 mL⋅kg^-1^⋅min^-1^) had microbiopsies taken from M. vastus lateralis and M. triceps brachii, which were analyzed for various measures of mitochondrial respiration using high-resolution respirometry. Thereafter, all participants tested $\dot{V}$O_2peak_ in both running (RUN) and upper body poling (UBP).

**Results:**

XC skiers had generally higher mitochondrial respiration in M. triceps brachii compared to CON (*P* < 0.001), whereas no significant group-differences in mitochondrial respiration in M. vastus lateralis were revealed. XC skiers had higher mitochondrial respiration in M. triceps brachii compared to M. vastus lateralis (*P* = 0.005–0.058), whereas in CON, most mitochondrial respiration measures were higher in M. vastus lateralis than in M. triceps brachii (*P* < 0.01). When all athletes were pooled, there was a strong positive correlation between $\dot{V}$O_2peak_ in UBP and mitochondrial respiration in M. triceps brachii on several measures (*P* < 0.01), whereas no correlation was found for RUN.

**Conclusion:**

The higher mitochondrial respiration found in M. triceps brachii compared to M. vastus lateralis among our elite XC skiers demonstrates the potential for the arm muscles to adapt to aerobic endurance training. The opposite pattern found in CON, clearly showed lower mitochondrial respiration in M. triceps brachii compared to XC skiers, whereas respiration in M. vastus lateralis did not differ between groups. The strong positive correlation between mitochondrial respiration in M. triceps brachii and $\dot{V}$O_2peak_ in UBP indicate that arm muscles’ respiratory function may be a limiting factor for $\dot{V}$O_2peak_ in arm-dominant exercise modes.

## Introduction

In endurance sports, performance is largely determined by the ability to produce and utilize energy aerobically. The upper limit for aerobic energy production in endurance events is maximal oxygen uptake ($\dot{V}$O_2max_) (Bransford and Howley, 1976; Bassett and Howley, 1997). Although the capacity of mitochondrial respiration is in excess of O_2_ supply, and $\dot{V}$O_2max_ is primarily limited by the delivery of O_2_ to the working muscles (Richardson, 2000; Boushel et al., 2011), the mitochondria’s capacity to utilize oxygen determines the oxygen demand of skeletal muscles during exercise (Hoppeler and Flueck, 2003; Turcotte, 2003). In this context, a strong relationship between $\dot{V}$O_2max_ and mitochondrial content and quality has been shown (Holloszy and Coyle, 1984; Tonkonogi and Sahlin, 2002; Jacobs and Lundby, 2013). In addition, the primary limitation to $\dot{V}$O_2max_ may differ between exercise modes, and e.g., the reliance on O_2_-utilization with a relatively small amount of exercising muscle mass (e.g., arm cranking) is shown to be higher than for leg and whole-body exercise (Boushel et al., 2011). This is explained by the lower systemic blood flow leading to sufficiently high blood flow per unit muscle mass (Boushel and Saltin, 2013). Furthermore, $\dot{V}$O_2max_ appears to be unaffected when O_2_-supply is further increased during exercise with a small amount of muscle mass (Pedersen et al., 1999; Hopman et al., 2003; Mourtzakis et al., 2004). Since the total muscle mass of the arms only equals about 40% of the leg muscle mass in trained populations with equally trained limbs (van Hall et al., 2003; Andersson et al., 2010; Carlsson et al., 2014), exercise modes primarily reliant on the arm muscles for propulsion may have a larger reliance on O_2_-utilization than modes primarily driven by the legs. For example, a previous study has shown that mitochondrial capacity does not exceed maximal O_2_-delivery for M. deltoideus in arm cycling (Boushel et al., 2011).

Besides these potential differences in the limitations to $\dot{V}$O_2max_, many other physiological responses are shown to differ between arm/upper-body and leg/whole-body exercise. For example, the $\dot{V}$O_2_- and heart rate (HR)-kinetics during arm exercise is slower (Koppo et al., 2002; Schneider et al., 2002) and blood lactate concentrations higher than for leg exercise at the same relative intensity (Mittelstadt et al., 1995; van Hall et al., 2003). Furthermore, O_2_-extraction is lower in arm than leg muscles (Calbet et al., 2005), which may be related to differences in muscle mitochondrial capacity (Cardinale et al., 2018a). In general, oxidative capacity and the content of type I muscle fibers in arm muscles are regarded to be lower compared to leg muscles (Johnson et al., 1973; Essén et al., 1975), with corresponding lower mitochondrial enzyme activities (Essén et al., 1975; Ara et al., 2011; Helge et al., 2011). However, the leg muscles of the participants examined in these studies are used more frequently than arm muscles, both during training and in everyday life, and therefore the training status is usually higher in legs than arms. Accordingly, this might have influenced the scientific comparisons of arm and leg muscles done to date.

To compare muscular adaptations without the limiting factor of different training status of arms and legs, elite XC skiers are valid participants displaying almost equally well-trained muscles of upper and lower limbs (van Hall et al., 2003; Holmberg, 2015). Among XC skiers, the leg muscles have shown a relatively higher content of type I muscle fibers and greater fat oxidation capacity compared to arm muscles, whereas the mitochondrial content seems to be equal across arms and legs (Ørtenblad et al., 2018). However, an increased quantity of mitochondria does not necessarily translate to improved function of the mitochondria, and mitochondrial respiration may increase without a concurrent increase in mitochondrial quantity (Yan et al., 2011; Yan et al., 2012). Therefore, it has been suggested that measurements of mitochondrial quantity should be combined with measurements of mitochondrial quality/function to provide a more comprehensive understanding. In this context, mitochondrial respiration, measured by use of High-Resolution Respirometry (HRR), has been used as a reference value of a muscle’s oxidative capacity (Larsen et al., 2012). Currently, the previous comparisons of arm and leg muscles done with HRR are limited by different training status of arms and legs and have, thus, demonstrated higher mitochondrial respiration in leg muscles (Boushel et al., 2011).

By comparing XC skiers and physically active controls (CON) represented by a population following the international exercise guidelines (WHO, 2010), our primary aim was to investigate differences in mitochondrial respiration (using HRR) between M. triceps brachii (arm) and M. vastus lateralis (leg). Our hypothesis was that mitochondrial respiration would be superior in M. vastus lateralis compared to M. triceps brachii for both groups and that mitochondrial respiration would be greater in XC skiers than CON, in particular for M. triceps brachii. The secondary aim was to investigate associations between mitochondrial respiration of arm and leg muscles and peak oxygen uptake ($\dot{V}$O_2peak_) in arm- and leg-dominant exercise modes. We hypothesized that a significant correlation between both $\dot{V}$O_2peak_ in upper body exercise and mitochondrial respiration of the M. triceps brachii and $\dot{V}$O_2peak_ in lower body exercise and mitochondrial function of M. vastus lateralis would be present.

## Materials and Methods

### Participants

Thirteen male elite XC skiers and twelve male CON voluntarily took part in the present study (characteristics in Table 1). Inclusion criteria for XC skiers was set to $\dot{V}$O_2peak_ above 70 mL⋅min^-1^ kg^-1^ in diagonal roller skiing, and participation in World Cup races, or other high ranked FIS-races in the 2015/2016 season. In CON, the criteria for participation was to exercise regularly according to the current national and international guidelines on physical activity (150 min of moderate intensity/week or 75 min of vigorous intensity) (WHO, 2010). The study was approved by the Norwegian Data Protection Authority. All subjects gave written informed consent in accordance with the Declaration of Helsinki.

**Table 1:** Anthropometric and physiological characteristics of the elite cross-country (XC) skiers and the physically active control group (CON) of this study (means ± SD).

Participants	XC skiers	CON
	(*n* = 13)	(*n* = 12)
Age (years)	25 ± 4	26 ± 3
Height (cm)	182 ± 3	184 ± 4
Mass (kg)	77 ± 4*	83 ± 9
Body fat (%)	10.4 ± 1.7*	14.3 ± 3.5
Total lean body mass (kg)	67.5 ± 3.9	69.4 ± 7.4
Arm lean mass (kg)	8.2 ± 0.5	8.5 ± 1.1
Leg lean mass (kg)	21.8 ± 2.0	23.8 ± 3.2
$\dot{V}$O_2peak_ RUN (mL⋅min^-1^⋅kg^-1^)	75.5 ± 4.2**	57.2 ± 6.4
$\dot{V}$O_2peak_ UBP (mL⋅min^-1^⋅kg^-1^)	55.1 ± 6.1**	35.8 ± 4.6

*Peak oxygen uptake in running (*$\dot{V}$O*_*2peak*_ RUN), peak oxygen uptake in upper body poling (*$\dot{V}$O*_*2peak*_ UBP). Mass (kg) is reported as a mean from three different assessments. ^∗, ∗∗^ Significantly different from CON at *p* < 0.05 and *p* < 0.01, respectively.*

### Study Design

All participants completed three testing sessions on separate days, and they were instructed not to engage in any vigorous physical activity at the days of testing. Participants performed two incremental exercise tests to exhaustion [running (RUN) and upper body poling (UBP)] on two separate days in a randomized order, to determine $\dot{V}$O_2peak_ and corresponding peak physiological responses. The third day of testing consisted of muscle biopsies as described in detail below and body composition assessment using dual-energy X-ray absorptiometry (DEXA).

### Exercise Tests

Both RUN and UBP began with a 10-min warm-up at an intensity of 6–8 rating of perceived exertion (RPE), followed by five to seven 5-min familiarization stages gradually increased with 1 km h^-1^ (RUN) and 20 W (UBP) for every stage. Blood lactate concentrations (BLa) and RPE were determined after every stage, and the warm-up was concluded when participants had BLa around 6 mmol L^-1^. Following warm-up and familiarization, participants had an active rest period of 10–15 min before the maximal test for attainment of $\dot{V}$O_2peak_ began. The participants began the maximal test at the same speed/power output at which they had an RPE of 12 during warm-up. Speed/power output increased every minute by 1 km h^-1^ (RUN) or 20 W (UBP) each minute until failure (4–7 min). Criteria for attainment of $\dot{V}$O_2peak_ was a leveling off in O_2_-uptake, respiratory exchange ratio (RER) > 1.05 or when a further increase in speed/power output was impossible. RER was calculated as the ratio of volume of exhaled carbon dioxide (CO_2_) and volume of inspired oxygen (O_2_). $\dot{V}$O_2peak_ was reported as the mean of the three highest 10 s values during the last minute of the test.

RUN was performed on a Forcelink 5 × 3 m treadmill (Forcelink, Zwolle, Netherlands) whereas UBP was performed on a modified Concept2 SkiErg (Morrisville, VT, United States) with the damper set at the middle drag setting and was adjusted for seated poling as previously described (Hegge et al., 2014). To minimize lower body involvement in the UBP movement, participants sat on an elevated bench in front of the SkiErg. Participants were fixed to the bench via straps around the pelvis and knees, this allowed free motion of the upper body whilst minimizing lower body contribution. Previous research have shown negligible activation in leg muscles when performed accordingly (Hegge et al., 2015). Ventilatory variables were measured and recorded continuously with Jaeger Oxycon pro with mixing chamber (Jaeger GmbH, Hoechberg, Germany). The instrument was calibrated against ambient air and commercial gas (Riessner Gase, Lichtenfels, Germany) with known concentrations of O_2_ (16.00%) and CO_2_ (5.85%) before each test session. The O_2_ and CO_2_ concentrations of room-air were measured and the flow transducer was calibrated using a 3-L High-precision calibration syringe (Calibration syringe D, SensorMedics, Yorba Linda, CA, United States). Heart rate (HR) was continuously recorded with a Polar m400 (Kempele, Finland). Rate of Perceived Exertion (RPE) using the Borg Scale (Borg, 1970), for total, ventilatory and muscular effort, and 20 μL of blood was drawn directly after the maximal test for the assessment of BLa using Biosen C-Line Sports lactate measurement system (EKF Industrial Electronics, Magdeburg, Germany). The Biosen device was calibrated every 60-min with a 12 mmol L^-1^ standard concentration.

### Tissue Handling and Preparation

Muscle biopsies were collected from M. vastus lateralis and the lateral head of M. triceps brachii and sampled in a randomized order from the participants left or right side. Although fiber type distribution likely differ between these muscles, not assessed in the present study, they were chosen due to their similar activation in double poling (Holmberg et al., 2005; Ørtenblad et al., 2018). To minimize discomfort for the participants a microbiopsy technique was used to collect muscle tissue (Hayot et al., 2005). Sampling site was carefully marked out on both muscles and the area was shaved and sterilized (chlorhexidine 5%). Sampling site was injected with a local anesthesia (xylocaine 2%, AstraZeneca, Oslo, Norway) prior to obtainment of muscle tissue. Skin was punctured with a 15-gauge co-axial introducer needle (BioPince, Medical Device Technologies Inc., Gainesville, FL, United States) and with a 16-gauge biopsy needle placed in the biopsy device the needle was placed through the co-axial intro needle and the muscle sample was obtained. Muscle sample was removed from the needle using two sterile forceps and placed in 2 ml ice-cold biopsy preservation solution (BIOPS) containing 2.77 mM CaK_2_EGTA, 7.23 mM K_2_EGTA, 5.77 mM Na_2_ATP, 6.56 mM M_g_Cl_2_ 6 H_2_O, Taurine, 15 mM Na_2_Phosphocreatine, 20 mM Imidiazole, 0.5 mM Dithiothreitol and 50 mM MES hydrate (Pesta and Gnaiger, 2012). Biopsy procedure was repeated two to four times until sufficient muscle tissue had been collected which was determined visually. Biopsy rested in BIOPS until dissection which was done the same day.

Fiber bundles were dissected using forceps under a microscope (Stereomicroscope Stemi 2000, Zeiss, Thornwood, NY, United States) to remove connective tissue and fat. Remaining muscle fibers were then chemically permeabilized via incubation in 2 ml BIOPS together with 50 μg mL^-1^ of saponin for 20-min in the fridge with mild shaking (Kunz et al., 1993). To remove saponin from the fiber bundles they were washed with a mitochondrial respiration medium (MiR05) (Pesta and Gnaiger, 2012) for 10-min at 4°C. Wet weight of the muscle bundles (2–3 mg) was measured in a scale (SemiMicro Balance ME235P, Sartorius, Göttingen, Germany) after it had been blotted dry with 5 layers of microscope paper (Linsenpapier, Karl Hecht, GmbH, Sondheim, Germany). For wet weight measurements in the present study coefficient of variation (CV) was 33%.

### Mitochondrial Measurements

For mitochondrial respiration measurements high-resolution Oxygraph-2k (Oroboros Instruments, Innsbruck, Austria) was used. All respiratory measurements of fiber bundles, with unknown fiber type distribution, were done in 2 ml of MiR05 at physiological 37°C, maintained constant ± 0.001°C. Oxygen concentrations in the chamber were kept between 200 and 500 μM for all experiments to avoid oxygen limitation (Pesta and Gnaiger, 2012). Oxygen flux was continuously recorded online using DatLab 6.1, allowing non-linear changes in the negative time derivative of the oxygen concentration signal and reported as tissue mass-specific respiration; per second, per milligrams of wet weight of muscle fibers (pmol⋅ s^-1^⋅mg^-1^). The CV was 29% for mitochondrial respiration measurements in the present study.

A specific Substrate, Uncoupler and Inhibitor Titration (SUIT) protocol was applied to determine individual aspects of respiratory control as previously described (Jacobs et al., 2011; Pesta and Gnaiger, 2012). When sufficient (>5 mg) muscle tissue had been collected respiratory measurements were made in duplicates. Forty-four percentage of respiratory measurements were made in duplicates (33% for M. vastus lateralis and 54% for M. triceps brachii). On average, 3.3 mg muscle tissue was added to the chambers (3.1 mg for M. vastus lateralis and 3.5 mg for M. triceps brachii). The SUIT protocol is described elsewhere (Pesta and Gnaiger, 2012). Titrations were added in seven steps as presented below (Supplementary Figure S1). Malate (2 mM) and octanoyl carnitine (0.2 mM) to induce leak respiration in absence of adenylates (L_N_), ADP (5 mM) to assess maximal electron flow through electron transferring-flavoprotein (ETF) and fatty acid oxidative capacity (P_ETF_). Further, glutamate (10 mM) was added to assess respiration specific to Complex I (P_CI_) followed by succinate (10 mM) to stimulate respiration through complex I and complex II (P_CI+II_) which is the maximal oxidative phosphorylation (OXPHOS) capacity. Step-wise addition of carbonyl cyanide m-chlorophenyl hydrazon (CCCP) (0.5 μM steps) to assess electron transfer system capacity (E_CI+CII_). Rotenone (0.5 μM) to inhibit complex I and finally malonic acid (5 mM) and antimycin A (2.5 μM) to inhibit complex II and complex III to determine the residual oxygen consumption (ROX).

As an internal normalization of flux, the OXPHOS coupling efficiency (j_≈P_) and the excess electron transfer system-phosphorylation capacity factor (j_ExP_) were calculated. j_≈P_ is calculated using the formula (P_CI+CII_ - L_N_)/P_CI+II_ = 1 - L_N_/P_CI+CII_) and represents free divided by total OXPHOS capacity, a lower value means less efficient coupling (Gnaiger, 2014). j_ExP_ is calculated with the formula (1 - P_CI+CII_/E_CI+CII_) and reflects what control the phosphorylation system have over OXPHOS capacity. The relative contribution of P_ETF_ toward maximal respiration (E_CI+CII_) was also assessed to investigate any potential differences in relative fat oxidation between and within groups (1 - P_ETF_/E_CI+CII_).

### Body Composition

Body composition was assessed by DEXA using whole body fan beam technology (Discovery A, Hologic, Marlborough, MA, United States). DEXA scan procedure is described in more detail elsewhere (Visser et al., 1999). Whole body values were presented as total mass (kg), relative percentage of fat (%) and lean body mass (LBM) (g). The sum of LBM in the right and left arm was presented as ArmLBM (g) and sum of lean body mass of right and left leg as LegLBM (g).

### Statistical Analysis

Normal distribution was assessed by Shapiro–Wilk’s test, and data are reported as means ± standard deviation. Comparisons of mitochondrial respiration, $\dot{V}$O_2peak_ and body composition were done using a two-way mixed ANOVA. When a significant main effect of interaction was observed it was further analyzed with univariate analysis. CV for mitochondrial respiration and wet weight measurements in the present study was also calculated [(SD⋅mean^-1^) ⋅ 100%]. Correlation between $\dot{V}$O_2peak_ and mitochondrial respiration was analyzed using Pearson’s product-moment correlation both with all participants pooled, and for both groups individually. All analyses were performed using IBM SPSS 24.0 program for Windows (Chicago, IL, United States) and level of significance was set at an alpha level < 0.05.

## Results

### Participant Characteristics

There were no significant differences between XC skiers and CON in age, height, LBM, ArmLBM and LegLBM. XC skiers had a 7% lower body mass and 3.9% lower body fat compared to CON (*P* < 0.05). XC skiers also had a significantly higher $\dot{V}$O_2peak_ for both UBP (35%) and RUN (23%) (*P* < 0.01) (Table 1).

### Mitochondrial Respiration

There was a significant interaction between group (XC skiers vs. CON) and muscle (M. vastus lateralis vs. M. triceps brachii) on CI-linked respiration *(P* < 0.005), maximal tissue mass-specific OXPHOS with combined CI+CII substrates (*P* < 0.005), uncoupled CI+CII linked respiration (*P* < 0.005) and uncoupled CII-linked respiration (*P* < 0.005*)*.

**FIGURE 1:**
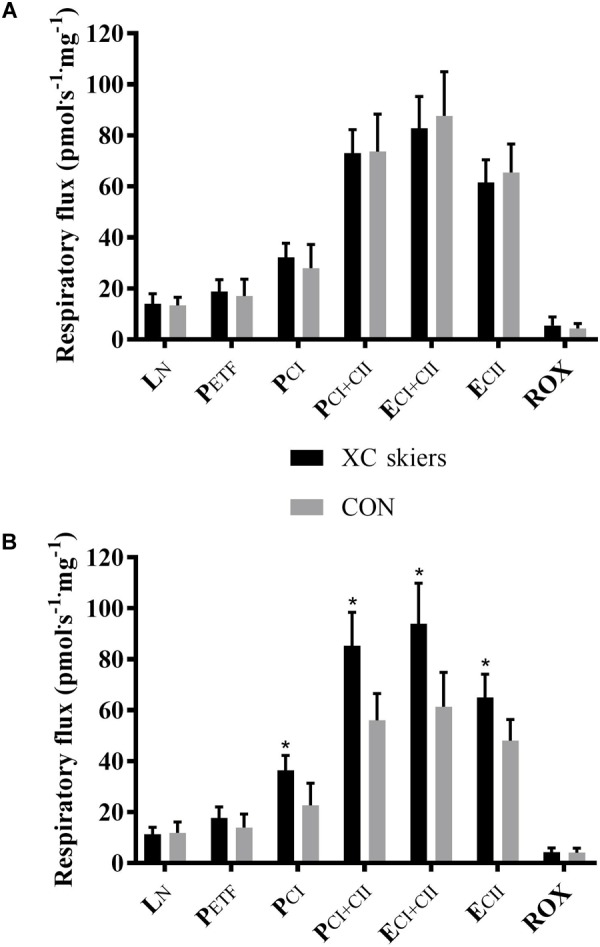
Differences in respiratory flux in M. triceps brachii and M. vastus lateralis between elite cross-country skiers (XC skiers) and physically active controls (CON). **(A)** M. vastus lateralis and **(B)** M. triceps brachii. Respiratory flux expressed as mass-specific respiratory capacity (pmol⋅s^-1^⋅mg^-1^) for leak respiration in absence of adenylates (L_N_), fatty acid oxidative capacity (P_ETF_), complex I respiration (P_CI_), complex I and II respiration combined (P_CI+CII_), electron transfer system capacity (E_CI+CII_), electron transfer system capacity of complex II alone (E_CII_) and residual oxygen consumption (ROX). Data are displayed as means ± SD. ^∗^Significant difference between groups (*P* < 0.001).

For M. vastus lateralis there was no significant difference between XC skiers and CON at any of the respiratory states (Figure 1A and Table 2). For M. triceps brachii XC skiers had significantly higher respiration rates compared to CON for P_CI_ (36.3 ± 5.9 vs. 22.6 ± 8.7 pmol⋅ s^-1^⋅mg^-1^) (*P* < 0.001), P_CI+CII_ (85.9 ± 13.3 vs. 56.1 ± 10.5 pmol⋅ s^-1^⋅mg^-1^) (*P* < 0.001), E_CI+CII_ (94.0 ± 16.6 vs. 61.3 ± 13.5 pmol⋅ s^-1^⋅mg^-1^) (*P* < 0.001) and E_CII_ (65.0 ± 9.1 vs. 48.1 ± 8.2 pmol⋅ s^-1^⋅mg^-1^) (*P* < 0.001) (Figure 1B and Table 2). Even though not reaching statistical significance, there was a trend toward higher respiration for XC skiers in M. triceps brachii compared to CON for P_ETF_ (*P* = 0.081).

**Table 2:** Mitochondrial respiratory flux in M. vastus lateralis and M. triceps brachii of the elite cross-country (XC) skiers and the physically active control (CON) group of this study (means ± SD).

	XC skiers		CON	
	M. vastus	M. triceps	M. vastus	M. triceps
	lateralis	brachii	lateralis	brachii
L_N_	14.3 ± 4.0	11.3 ± 2.9	13.4 ± 3.2	11.8 ± 4.3
P_ETF_	19.1 ± 4.7	17.9 ± 4.5	17.1 ± 6.6	14.0 ± 5.3
P_CI_	32.0 ± 5.6	36.3 ± 5.9^#^	28.0 ± 9.3	22.6 ± 8.7*
P_CI+CII_	73.1 ± 9.2	85.9 ± 13.3*^#^	73.7 ± 14.6	56.1 ± 10.5*
E_CI+CII_	82.7 ± 12.5	94.0 ± 16.6^#^	87.6 ± 17.3	61.3 ± 13.5*
E_CII_	61.6 ± 8.8	65.0 ± 9.1^#^	65.5 ± 11.1	48.1 ± 8.2*
ROX	5.5 ± 3.4	4.2 ± 1.8	4.4 ± 1.8	4.1 ± 1.8

*Respiratory flux expressed as mass-specific respiratory capacity (pmol⋅s^-*1*^⋅mg^-*1*^) for leak respiration in absence of adenylates (L_*N*_), fatty acid oxidative capacity (P_*ETF*_), complex I respiration (P_*CI*_), complex I and II respiration combined (P_*CI*+*CII*_), electron transfer system capacity (E_*CI*+*CII*_), electron transfer system capacity of complex II alone (E_*CII*_) and residual oxygen consumption (ROX). ^∗^Significantly different from M. vastus lateralis, #significantly different from CON at *p* < 0.05.*

**FIGURE 2:**
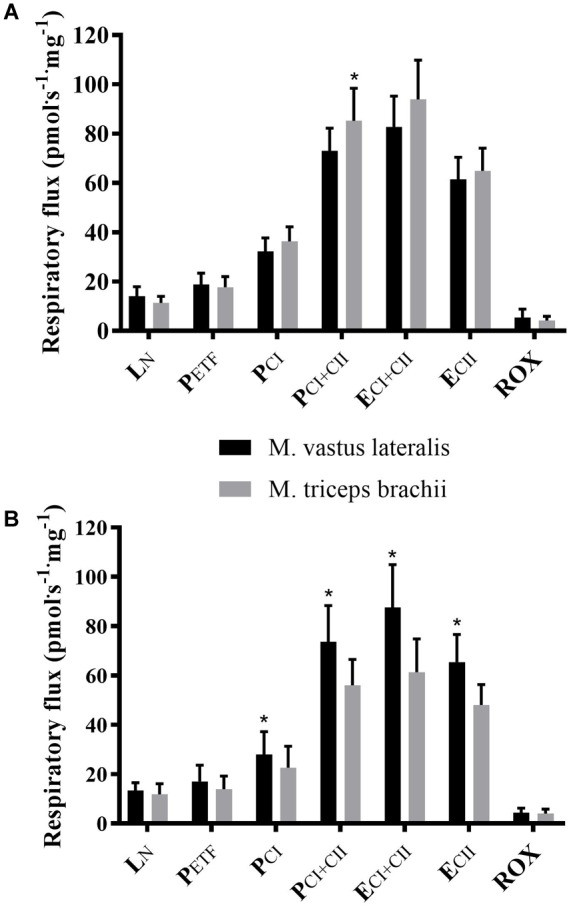
Differences in respiratory flux between M. triceps brachii and M. vastus lateralis in XC skiers and CON. **(A)** elite cross-country skiers (XC skiers) and **(B)** physically active controls (CON). Respiratory flux expressed as mass-specific respiratory capacity (pmol⋅s^-1^⋅mg^-1^) for leak respiration in absence of adenylates (L_N_), fatty acid oxidative capacity (P_ETF_), complex I respiration (P_CI_), complex I and II respiration combined (P_CI+CII_), electron transfer system capacity (E_CI+CII_), electron transfer system capacity of complex II alone (E_CII_) and residual oxygen consumption (ROX). Data are displayed as means ± SD. ^∗^Significant difference between muscles (*P* < 0.001).

Within groups, XC skiers’ mass specific mitochondrial respiration was significantly higher in M. triceps brachii compared to M. vastus lateralis for P_CI+CII_ (85.9 ± 13.3 vs. 73.1 ± 9.2 pmol⋅ s^-1^⋅mg^-1^) (*P* = 0.005) (Figure 2A and Table 2). Although not reaching statistical significance there was also a strong trend toward higher respiration in M. triceps brachii compared to M. vastus lateralis within XC skiers for E_CI+CII_ (94.0 ± 16.6 vs. 82.7 ± 12.5 pmol⋅ s^-1^⋅mg^-1^) (*P* = 0.058). In CON respiration was significantly higher for M. vastus lateralis compared to M. triceps brachii for P_CI_ (28.0 ± 9.3 vs. 22.6 ± 8.7 pmol⋅ s^-1^⋅mg^-1^) (*P* = 0.002), P_CI+CII_ (73.7 ± 14.6 vs. 56.1 ± 10.5 pmol⋅ s^-1^⋅mg^-1^) (*P* = 0.002), E_CI+CII_ (87.6 ± 17.3 vs. 61.3 ± 13.5 pmol⋅ s^-1^⋅mg^-1^) (*P* = 0.001) and E_CII_ (65.5 ± 11.1 vs. 48.1 ± 8.2) (*P* = 0.001) (Figure 2B and Table 2).

**FIGURE 3:**
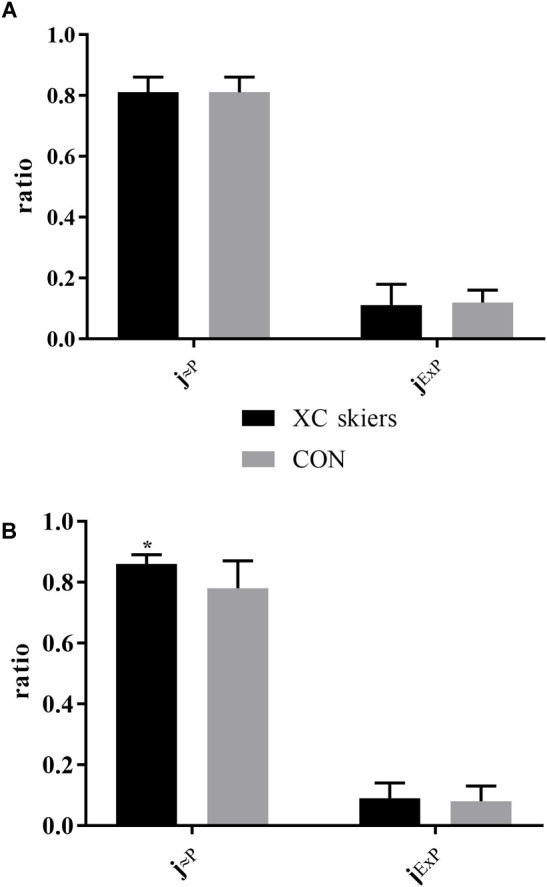
Differences in respiratory control ratios in M. triceps brachii and M. vastus lateralis between elite cross-country skiers (XC skiers) and physically active controls (CON). **(A)** M. vastus lateralis and **(B)** M. triceps brachii. Mitochondrial quality noted as ratio between 0 and 1 for oxidative phosphorylation capacity efficiency (j_≈P_) and excess electron transfer system-phosphorylation capacity factor (j_ExP_). Data are displayed as means ± SD. ^∗^Significant difference between groups (*P* < 0.001).

**FIGURE 4:**
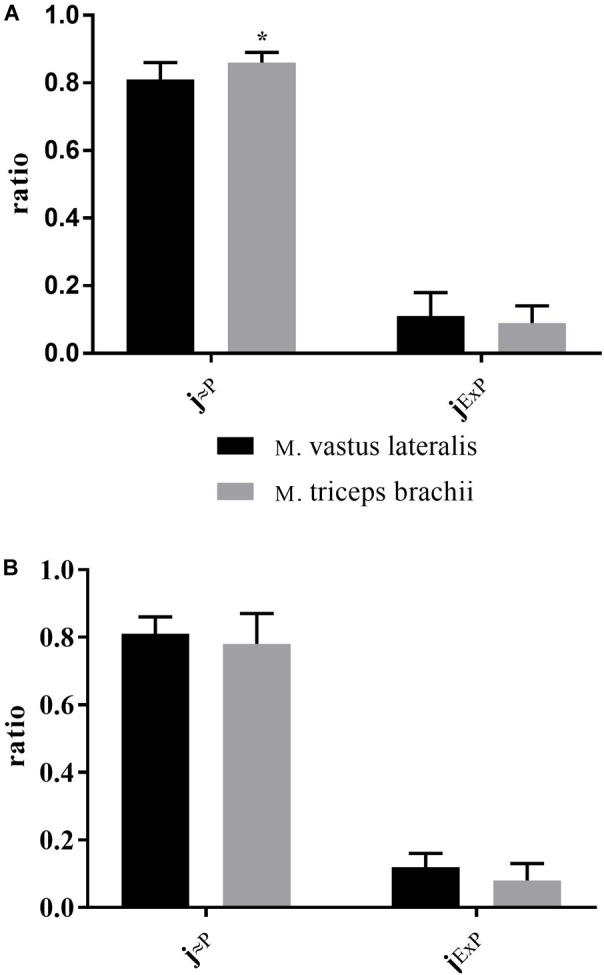
Differences in respiratory control ratios between M. triceps brachii and M. vastus lateralis in XC skiers and CON. **(A)** elite cross-country skiers (XC skiers) and **(B)** physically active controls (CON). Mitochondrial quality noted as ratio between 0 and 1 for oxidative phosphorylation capacity efficiency (j_≈P_) and excess electron transfer system-phosphorylation capacity factor (j_ExP_). Data are displayed as means ± SD. ^∗^Significant difference between groups (*P* < 0.001).

There was a significant difference between XC skiers and CON for j_≈P_ in M. triceps brachii (*P* = 0.006) (Figure 3B) but not in M. vastus lateralis (Figure 3A). For j_ExP_ there was no difference between groups for the two muscles (Figure 3A,B). In XC skiers j_≈P_ was significantly better in M. triceps brachii compared to M. vastus lateralis (*P* = 0.007) but no difference in j_ExP_ (Figure 4A). Within CON there was no difference for either j_≈P_ or j_ExP_ (Figure 4B). Contribution of P_ETF_ to E_CI+CII_ was significantly higher for M. vastus lateralis compared to M. triceps brachii within XC skiers (23.2 ± 5.6 vs. 19.5 ± 3.5 %) (*P* = 0.036). In addition, there was a trend to greater relative contribution of P_ETF_ in M. vastus lateralis for XC skiers compared to CON (23.2 ± 5.6 vs. 18.2 ± 7.2 %) (*P* = 0.091).

### Correlation Between $\dot{V}$O_2peak_ and Mitochondrial Respiration

**FIGURE 5:**
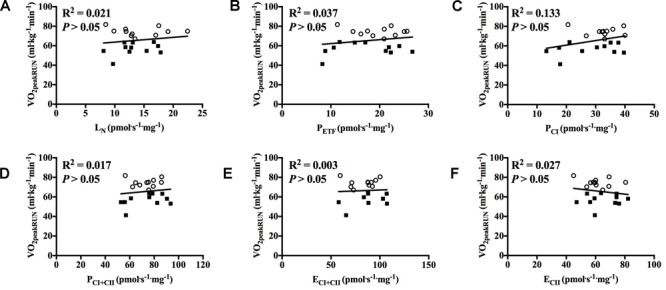
Correlation between peak oxygen uptake (VO_2peak_) in running and respiratory flux for M. vastus lateralis with participants pooled. **(A)** leak respiration in absence of adenylates (L_N_), **(B)** fatty acid oxidative capacity (P_ETF_), **(C)** complex I respiration (P_CI_), **(D)** complex I and complex II respiration combined (P_CI+CII_), **(E)** electron transfer system capacity (E_CI+CII_), and **(F)** electron transfer system capacity of complex II alone (E_CII_). ■ = CON, ○ = XC skiers.

**FIGURE 6:**
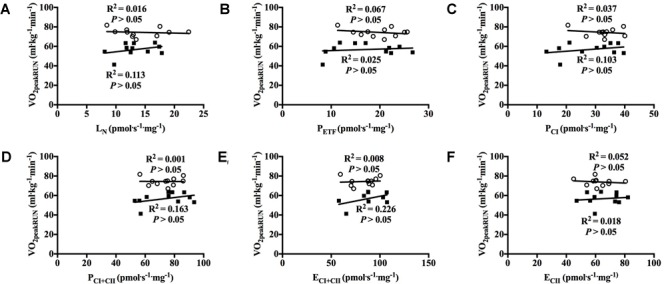
Correlation between peak oxygen uptake (VO_2peak_) in running and respiratory flux for M. vastus lateralis with both groups individually. **(A)** leak respiration in absence of adenylates (L_N_), **(B)** fatty acid oxidative capacity (P_ETF_), **(C)** complex I respiration (P_CI_), **(D)** complex I and complex II respiration combined (P_CI+CII_), **(E)** electron transfer system capacity (E_CI+CII_) and **(F)** electron transfer system capacity of complex II alone (E_CII_). ■ = CON, ○ = XC skiers.

With all participants pooled and when each group was examined separately there was no significant correlation between mitochondrial respiration in any of the different respiratory states for M. vastus lateralis and $\dot{V}$O_2peak_ in RUN (Figure 5A–F, 6A–F).

**FIGURE 7:**
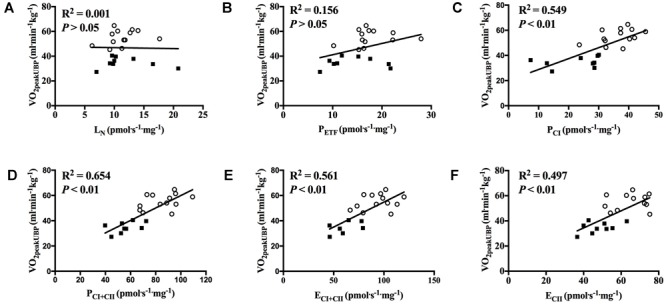
Correlation between peak oxygen uptake (VO_2peak_) in upper body poling and respiratory flux for M. triceps brachii with participants pooled. **(A)** leak respiration in absence of adenylates (L_N_), **(B)** fatty acid oxidative capacity (P_ETF_), **(C)** complex I respiration (P_CI_), **(D)** complex I and complex II respiration combined (P_CI+CII_), **(E)** electron transfer system capacity (E_CI+CII_), and **(F)** electron transfer system capacity of complex II alone (E_CII_). ■ = CON, ○ = XC skiers.

**FIGURE 8:**
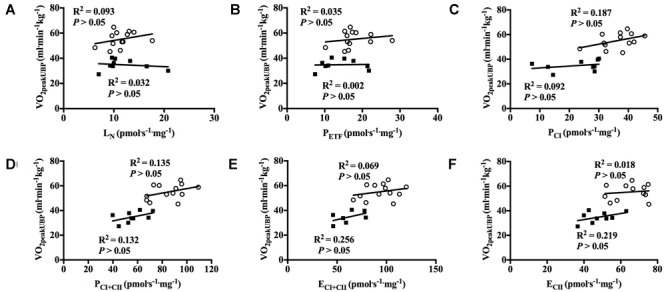
Correlation between peak oxygen uptake (VO_2peak_) in upper body poling and respiratory flux for M. triceps brachii with both groups individually. **(A)** leak respiration in absence of adenylates (L_N_), **(B)** fatty acid oxidative capacity (P_ETF_), **(C)** complex I respiration (P_CI_), **(D)** complex I and complex II respiration combined (P_CI+CII_), **(E)** electron transfer system capacity (E_CI+CII_), and **(F)** electron transfer system capacity of complex II alone (E_CII_). ■ = CON, ○ = XC skiers.

With all participants pooled, there was a strong positive correlation between $\dot{V}$O_2peak_ in UBP and P_CI_, P_CI+CII_, E_CI+CII_ and E_CII_ (all *P* < 0.01; Figure 7A–F). OXPHOS capacity assessed by combined CI+CII substrates (P_CI+CII_) had the strongest correlation, in which 65% of the variance in $\dot{V}$O_2peak_ could be explained by mitochondrial respiration in this state. When examined individually, the significant correlation between mitochondrial respiration for M. triceps brachii and $\dot{V}$O_2peak_ in UBP disappeared (Figure 8A–F).

## Discussion

The main findings of the present study were as follows: (1) XC skiers had higher mitochondrial respiration in M. triceps brachii but not in M. vastus lateralis compared to CON; (2) XC skiers had higher respiration in M. triceps brachii compared to M. vastus lateralis, whereas the opposite pattern was found for CON, and (3) when participants from both groups were pooled, several mitochondrial respiratory states in M. triceps brachii were significantly correlated with $\dot{V}$O_2peak_ in UBP but no states of respiration for M. vastus lateralis correlated with $\dot{V}$O_2peak_ in RUN.

### Mitochondrial Respiration

This is the first study to compare mitochondrial respiration using HRR in M. triceps brachii in humans between groups with different training status. Our novel findings of higher mitochondrial respiration in elite XC skiers compared to CON are in agreement with a previous study where an increase in arm mitochondrial enzyme activities were shown in athlete groups who regularly involve upper-body work in their training compared to athletes who mainly exercise with the legs (Gollnick et al., 1972). The current study extends these findings by investigating integrative mitochondrial respiration. Our finding that mitochondrial respiration for L_N_ and P_ETF_ were the same for both groups differs from a previous comparison between active and elite participants (Jacobs and Lundby, 2013). However, similar to our findings, previous studies have also demonstrated differences in OXPHOS capacity between differently trained populations without any differences in basal or resting respiration (L_N_) (Mettauer et al., 2001; Zoll et al., 2002). Although not reaching statistical significance, there was a strong trend toward higher P_ETF_ in the M. triceps brachii for our XC skiers. This finding indicates that the capacity for fat oxidation in the arms of elite XC skiers may be greater than for CON, which generates a hypothesis for further investigation with a larger sample size. For most of the population, the daily use of the upper body is relatively low compared to the use of the legs whereas XC skiers is an athlete group who perform high amounts of upper-body work (Mahood et al., 2001; Terzis et al., 2006; Sandbakk et al., 2011; Holmberg, 2015). This is the most likely explanation for the high mitochondrial respiration levels found in the arms of XC skiers. This was not the case for CON who, despite having a higher average $\dot{V}$O_2peak_ than the normal population (Loe et al., 2014), mainly participated in activities using leg musculature. Altogether, this explains the large difference observed in mitochondrial respiration for M. triceps brachii between XC skiers and CON in this study.

In contrast, no differences in mitochondrial respiration for M. vastus lateralis were found between XC skiers and CON. At first glance, our results appear to contrast previous studies that found greater mitochondrial respiration associated with improved aerobic fitness (Mettauer et al., 2001; Zoll et al., 2002; Daussin et al., 2008; Jacobs and Lundby, 2013). However, these previous results include some important nuances. For example, respiratory states excluding the electron input from P_CII_, mitochondrial respiration appear to be the same for both trained and untrained participants (Mettauer et al., 2001; Zoll et al., 2002; Daussin et al., 2008), although higher respiration have been reported in trained participants (Jacobs and Lundby, 2013; Dandanell et al., 2018). Furthermore, when comparing four different groups, ranging from trained to elite participants, differences were only found when comparing the least and best trained groups (Jacobs and Lundby, 2013). In comparison, both groups examined in our study were on a high level, and even CON closely matched the highly trained group of Jacobs and Lundby (2013) in terms of absolute $\dot{V}$O_2peak_ values (4.83 vs. 4.82 L⋅min^-1^). Our results also contrast previous findings that found higher P_ETF_ and P_CI+CII_ in XC skiers compared to untrained participants (Dandanell et al., 2018). However, the participants in CON in the present study had higher $\dot{V}$O_2peak_ than the untrained participants in the study by Dandanell et al. (2018) (57 vs. 48 mL⋅min^-1^ kg^-1^). The lack of difference in mitochondrial respiration for M. vastus lateralis between groups may imply that there is a ceiling level in respiration after which more training and a higher fitness level do not lead to higher mitochondrial respiration.

Contrary to our hypothesis, XC skiers had higher mitochondrial respiration for P_CI+CII_ and a strong trend toward higher respiration for E_CI+CII_ in M. triceps brachii compared to M. vastus lateralis. This was not the case in CON, where M. vastus lateralis had higher mitochondrial respiration than M. triceps brachii. Moreover, the findings in our XC skiers contrast with previous studies showing greater or similar mitochondrial content in leg vs. arm muscles in XC skiers (Mizuno et al., 1990; van Hall et al., 2003; Terzis et al., 2006; Ørtenblad et al., 2018). The mechanism behind the higher OXPHOS capacity of M. triceps brachii compared to M. vastus lateralis in XC skiers is not known. It is possible to speculate that the higher OXPHOS capacity of M. triceps brachii in XC skiers is due to a higher use of M. triceps brachii compared to M. vastus lateralis in their training. However, we find this unlikely due to several factors. Primarily, data collection for the present study was conducted in October-December, which comprise the end of preparation period and early competition period for XC skiers (Sandbakk and Holmberg, 2017). During the preparation period much of the performed training for XC skiers is running and cycling, which are both leg-dominant modes. In addition, both arms and legs are utilized during XC skiing and roller skiing, and even during double poling, which previously have been described as involving only upper body work, the activation and work of M. vastus lateralis is high and not much different from that of M. triceps brachii (Holmberg et al., 2005). Therefore, we speculate that higher mitochondrial respiration of M. triceps brachii in XC skiers is due to inherent differences between M. triceps brachii and M. vastus lateralis. A possible explanation could be related to mitochondrial O_2_ affinity (p50_mito_) and O_2_ extraction, where O_2_ extraction decrease as p50_mito_ increase (Cardinale et al., 2018a). p50_mito_ is higher and O_2_ extraction is lower as mitochondrial respiration approaches maximal respiratory capacity. Subsequently, when O_2_ delivery per active muscle mass is high, as for example in UBP, mitochondria respire closer to its maximal capacity, hence p50_mito_ is higher and O_2_ extraction lower. An increase in mitochondrial respiration in upper body may be an important adaptation to allow for increased O_2_ extraction with altered $\dot{V}$O_2peak_ in our XC skiers. However, the role of excess capacity of mitochondria of arm muscles and p50_mito_ warrants further investigation in future studies.

Another possible explanation is that the lower muscle mass of arms compared to legs results in a higher O_2_ delivery per active muscle mass in the arms of XC skiers during their regular training and that this constant exposure of a higher O_2_ delivery enhances the mitochondrial adaptations (Cardinale et al., 2018b). Finally, the higher mitochondrial respiration of M. triceps brachii compared to M. vastus lateralis in our XC skiers may be related to the higher amounts of lactate produced in the arms compared to the legs (van Hall et al., 2003). Increased lactate production may have a positive influence on mitochondrial adaptations as lactate infusion during exercise has been proposed as the cause of an increase in the expression of peroxisome proliferator-activated receptor-γ (PPAR-γ) coactivator-1α (PGC-1α) post-exercise (Hashimoto et al., 2007). PGC-1α is thought to be the key to mitochondrial biogenesis, and activation leads to a transcriptional process that ultimately yields increased mitochondrial quantity (Turcotte, 2003; Yan et al., 2011). Besides having a key role in mitochondrial biogenesis, PGC-1α has also been proposed as playing an important role in the qualitative alterations of mitochondria (Yan et al., 2012). Therefore, we speculate that in their regular training, elite XC skiers produce more lactate in their arms compared to their legs, and that this production drives mitochondrial adaptation in M. triceps brachii to a greater extent than in M. vastus lateralis. The opposite pattern found in CON, with higher mitochondrial respiration in M. vastus lateralis compared to M. triceps brachii, is likely due to lower use of arms than legs in their training.

OXPHOS coupling efficiency (j_≈P_) was significantly higher in XC skiers for M. triceps brachii compared to CON, and for M. triceps brachii compared to M. vastus lateralis within the group of XC skiers. This suggests that in M. triceps brachii for XC skiers there is less proton leak, indicating a more efficient oxidative phosphorylation of ADP to adenosine triphosphate (ATP). Although we did not observe any difference in mitochondrial respiration for P_ETF_ between M. triceps brachii and M. vastus lateralis in XC skiers, we found lower relative fat oxidation in M. triceps brachii compared to M. vastus lateralis. This indicate that despite an overall greater mitochondrial respiration of M. triceps brachii, the need for fat oxidation is lower than in M. vastus lateralis. This is in line with a previous study in a similar group of XC skiers showing reduced fat oxidation capacity of the arms compared to legs (Ørtenblad et al., 2018). Furthermore, a trend toward greater relative contribution of fat oxidation in M. vastus lateralis for XC skiers than CON was observed. Also this is in line with a previous study showing that at the same absolute intensity contribution of fat oxidation was higher in trained vs. untrained (van Loon et al., 1999).

### Correlation Between $\dot{V}$O_2peak_ and Mitochondrial Respiration

We observed a strong positive correlation between mitochondrial respiration in M. triceps brachii and $\dot{V}$O_2peak_ in UBP with all participants pooled. To our knowledge this is the first study to investigate the relationship between mitochondrial respiration in an upper body muscle and $\dot{V}$O_2peak_ in an arm-dominant exercise mode. However, our findings are confirmed by a previous study which showed higher mitochondrial respiration with improved aerobic power (Jacobs and Lundby, 2013), although that study investigated the correlation between $\dot{V}$O_2max_ during a leg-dominant exercise mode and mitochondrial respiration in M. vastus lateralis. We therefore speculate that a higher $\dot{V}$O_2peak_ in UBP is partly due to higher mitochondrial respiration of M. triceps brachii and to a lesser degree by increased O_2_ supply through central factors. Our speculations are confirmed by a previous study where mitochondrial function in the upper body M. deltoideus closely matched oxygen delivery during arm exercise in sedentary participants (Boushel et al., 2011), and therefore an increased respiratory capacity of mitochondria is necessary to obtain a higher $\dot{V}$O_2peak_ during arm-dominant exercise modes. Although there was a strong positive correlation between many of the mitochondrial respiration states in M. triceps brachii and $\dot{V}$O_2peak_ in UBP we found no correlation with P_ETF_, which further demonstrate that fat oxidation does not have a pivotal role for $\dot{V}$O_2peak_ in UBP. However, it should be noted that we found no significant correlation between mitochondrial respiration in M. triceps brachii and $\dot{V}$O_2peak_ in UBP when the two groups were examined separately, which is partly due to low sample size and more homogenous levels of fitness within the groups.

No significant correlation between mitochondrial respiration for M. vastus lateralis and $\dot{V}$O_2peak_ in RUN was revealed. The lack of a relationship between mitochondrial respiration and RUN $\dot{V}$O_2peak_ with all participants pooled in the present study is in line with the similar mitochondrial respiration between groups, despite a significantly higher $\dot{V}$O_2peak_ during RUN for XC skiers. Our findings contrast previous findings that have showed a significant correlation between $\dot{V}$O_2max_ in cycling and mitochondrial respiration with subject groups differing in aerobic fitness (Jacobs and Lundby, 2013). Although mitochondrial respiration increase in conjunction with increased $\dot{V}$O_2peak_ (Jacobs and Lundby, 2013), we speculate that the excess capacity of mitochondria is sufficient at a certain level of aerobic fitness. Thereafter, other factors would adapt to a greater extent and further distinguish $\dot{V}$O_2peak_ during exercise using a large muscle mass.

### Limitations

Although the microbiopsy technique is regarded as a valid technique for mitochondrial respiration measurements, it also has disadvantages compared to the use of the traditional Bergstrøm-needle. For example, due to the small muscle samples obtained here we were not able to measure mitochondrial quantity and thereby distinguish if the observed differences in mitochondrial function were due to an increased number of mitochondria or due to an increased quality of the mitochondria. Furthermore, the small muscle samples are more susceptible to damage during the preparation phase and respiration rates might therefore be underestimated (Isner-Horobeti et al., 2014). This tendency is confirmed by our findings where respiration rates for all states except L_N_ is lower compared to previous studies (Jacobs et al., 2013; Christensen et al., 2016). Another limitation of our design is that we did not assess the mitochondrial outer membrane with the cytochrome c control test during respiratory measurements. Therefore, we can not be certain that the outer membrane was intact during respiratory measurements. In addition, the mitochondrial respiration protocol used in the present study come with a few limitations. Due to low muscle wet weight and/or technical errors we do not have complete *N* for all mitochondrial measurements. Furthermore, we did not assess fiber type profiles of the different muscles and therefore have not taken this into account when interpreting the data, although fiber type distribution likely differ between muscles (Ørtenblad et al., 2018). Overall, we believe the main conclusions drawn here have taken these limitations into consideration and are thus valid to answer our purposes. Finally, to further understand differences in mitochondrial capacity between muscles of different training status, it would have been useful to include a control group at the lower end of the fitness spectrum or specially trained runners or cyclists in future studies.

## Conclusion

In the present study, we found higher mitochondrial respiration for M. triceps brachii in elite XC skiers compared to CON, while the corresponding values for M. vastus lateralis did not differ between the two groups. This indicates a large potential for the arm muscles to adapt to aerobic endurance training, which is further supported by the higher mitochondrial respiration found in M. triceps brachii compared to M. vastus lateralis among our elite XC skiers. The similar mitochondrial respiration of M. vastus lateralis across groups indicate that extensive use of the legs in daily life together with exercise according to the national and international guidelines is sufficient for optimizing the mitochondrial adaptations. Accordingly, differences in $\dot{V}$O_2peak_ in RUN between XC skiers and CON are mainly explained by cardiovascular factors rather than respiratory capacity in the working muscles.

We found a strong positive correlation between mitochondrial respiration in M. triceps brachii and $\dot{V}$O_2peak_ in UBP with all participants pooled, whereas the corresponding association between $\dot{V}$O_2peak_ in RUN and mitochondrial respiration in M. vastus lateralis was not significant. Although part of this relationship was explained by inclusion of two groups, it still indicates that the greater $\dot{V}$O_2peak_ in XC skiers compared to CON is partly explained by higher mitochondrial respiration.

## Author Contributions

JB, VU, ØS, and AT involved in the study design and analyzed the data. JB, VU, ØR-H, and LA collected the data. All authors interpreted the results, contributed to the drafting, and revised the manuscript.

## Conflict of Interest Statement

The authors declare that the research was conducted in the absence of any commercial or financial relationships that could be construed as a potential conflict of interest.

## Supplementary Material

FIGURE S1 Respiratory example of the SUIT protocol. Example of respirometric trace for a representative participant in the XC skier group for M. triceps brachii.
