# Supplementary figures and images for: Comparison of Mitochondrial Respiration in M. triceps brachii and M. vastus lateralis Between Elite Cross-Country Skiers and Physically Active Controls
Source: Front Physiol. 2019 Apr 5;10:365. doi: 10.3389/fphys.2019.00365 (PMC6461012; doi:10.3389/fphys.2019.00365)

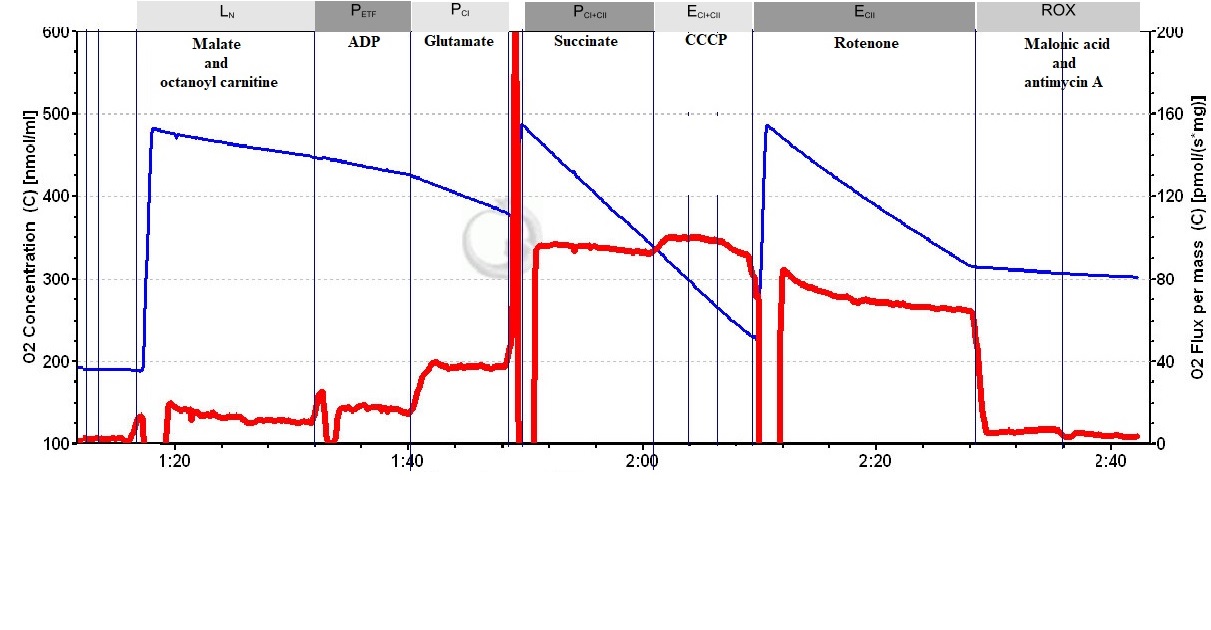

Supplement: FIGURE S1 — Respiratory example of the SUIT protocol. Example of respirometric trace for a representative participant in the XC skier group for M. triceps brachii. [file Image_1.JPEG]
